# Quantifying subsurface fracture damage in glaciers using fiber-optic seismology

**DOI:** 10.1126/sciadv.aef1107

**Published:** 2026-07-23

**Authors:** Thomas S. Hudson, Fabian Walter, Sebastian Noe, Andrea Zunino, John-Michael Kendall, Pascal Edme, Andreas Fichtner

**Affiliations:** ^1^ Department of Earth and Planetary Sciences, ETH-Zürich, Zürich, Switzerland.; ^2^ Swiss Federal Research Institute WSL, Birmensdorf, Switzerland.; ^3^ Laboratory of Hydraulics, Hydrology and Glaciology VAW, ETH-Zürich, Zürich, Switzerland.; ^4^ Department of Earth Sciences, University of Oxford, Oxford, UK.

## Abstract

Crevassing critically controls glacier stability. Crevasses can penetrate deep into a glacier or ice shelf, promoting calving, ice avalanches, and even sudden catastrophic ice shelf collapse. Yet quantifying subsurface fracture damage remains largely unquantified. Here, we show how distributed acoustic sensing technology can quantify subsurface fracture damage in unprecedented detail at an alpine glacier. We first demonstrate that seismic anisotropy can quantify fracture extent. We also study crevasse icequake failure mechanisms, which fail predominantly via tensile opening. Icequake-derived crevasse opening is consistent with anisotropy-derived estimates (∼8% of total ice volume), suggesting that damage is dominated by fracture rather than melt. These results establish a scalable approach for monitoring subsurface ice damage that complements existing satellite surface observations. Applications range from monitoring the stability of alpine glaciers that pose a risk to alpine communities to providing foundations for assessing subsurface fracture extent at globally pertinent ice sheets and ice shelves.

## Teaser

Fiber-optic sensing quantifies subsurface glacier damage in unprecedented detail, complementing satellite surface imagery.

## INTRODUCTION

Subsurface glacier damage is hypothesized to play an important role in driving ice loss and future sea level rise (*1*, *2*). The near-surface tensile stresses required to open surface cracks, or crevasses, are driven by basal topography (*3*, *4*), basal crevasses (*5*), and meltwater ponding (*6*, *7*). In the case of hanging and calving glaciers, tensile stresses may also be driven by gravity at the glacier front. These tensile stresses drive surface crevassing that damages the ice fabric (*8*–*10*). In a melt-free environment, crevasse depths are limited by the balance of horizontal tensile stresses and vertical overburden pressure. However, if meltwater enters a crevasse fracture, then the additional pressure of the denser water can cause crevasses to undergo hydrofracture, propagating far deeper than otherwise possible. This can provide pathways for meltwater to reach the glacier bed and enhance basal sliding (*4*, *6*, *11*), increasing sea level rise at ocean-terminating glaciers (*12*). Hydrofracture can also cause the rapid disintegration of ice shelves that would, otherwise, resist grounded ice flowing into the oceans (*5*, *7*, *13*, *14*). Furthermore, even if meltwater does not drive hydrofracture, it can still flow through crevasses, enhancing subsurface melt-driven ice loss, or voids, that may be hidden at the surface (*15*–*17*). While surface observations from satellite and unmanned aerial vehicle imagery are readily available (*10*, *18*), quantifying subsurface crevasse damage mechanisms and extent remains challenging.

One of the few tools capable of elucidating the glacier subsurface is seismology. As crevasses fracture, they generate icequakes that can be used to directly interrogate fracture mechanisms. These crevasse icequakes typically also generate surface waves that can be used to image the subsurface structure. Previous studies have shown that crevasses typically exhibit tensile faulting (*19*–*22*) and fracture-driven seismic velocity anisotropy (*17*, *23*). Hydrofracture-driven crevassing is inferred implicitly from auxiliary observations, such as drainage coinciding with seismicity (*6*, *24*–*27*). Tentative direct observations have also been made (*9*). However, quantifying crevasse fracture mechanisms and damage extent has been limited by spatial density of seismic receivers. Practically deploying seismic sensors in crevasse fields is challenging, while the cost per channel of traditional instrumentation limits deployments to only tens of sensors. Denser sampling is required to adequately image sub-meter fractures over hundred-meter scales.

**Fig. 1.**
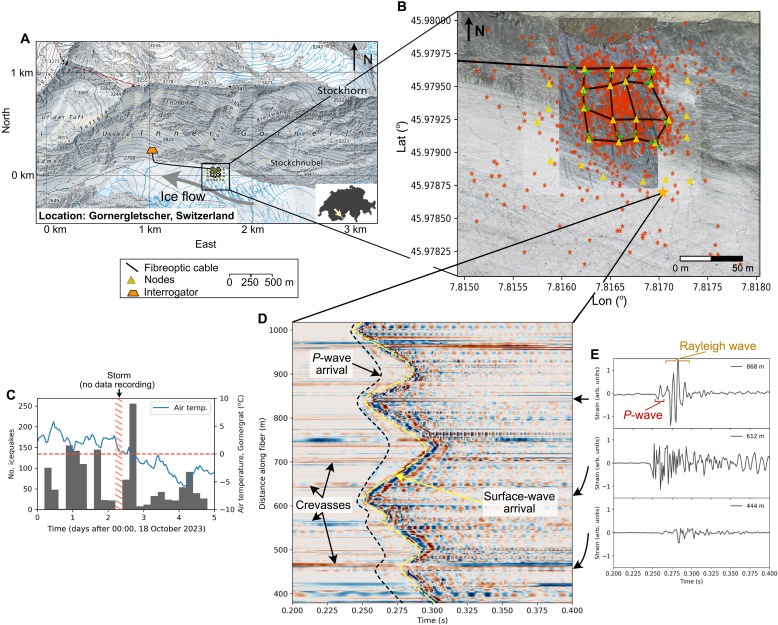
Experimental setup and detected icequakes. (**A**) Map of field site, showing fiber-optic interrogator, approximate fiber, and seismic nodes geometry. Background map obtained from the Swiss Federal Office of Topography. (**B**) Detailed map of crevasse study site, showing 2D fiber-optic grid (black line), nodes (yellow triangles), and crevasse seismicity (red stars). Green arrows indicate direction of the single continuous fiber from the interrogator. Detailed background imagery of section of the study site is from UAV imagery obtained during the deployment, overlaid on 2023 aerial imagery from the Swiss Federal Office of Topography (swisstopo). (**C**) Icequake occurrence rate through time compared to air temperature measured at Gornergrat, ∼500 m north of the study site. Temperature data are from the Swiss Federal Office of Meteorology and Climatology. Red dashed region indicates storm. (**D**) Example of an icequake arrival on the fiber-optic cable. *P*-wave and surface-wave arrivals, as well as decoupling due to the fiber traversing crevasses (labeled “Crevasses”) are labeled. Color map denotes normalized strain (red is positive and blue is negative). (**E**) Examples of waveforms at three DAS channels (with examples of *P*-wave and Rayleigh wave labeled).

Fortunately, recent technological advances, specifically fiber-optic sensing [or distributed acoustic sensing (DAS)], can now provide meter-resolution spatial sampling over crevasse-field scales. As well as providing far denser sampling than traditional seismic instrumentation, fiber-optic cables are faster and safer to deploy over active crevasse fields. Furthermore, only a single recording instrument (or interrogator) is required, enabling the potential for real-time monitoring. Here, we deployed a 1-km fiber-optic cable and 29 coincident seismic nodes in a two-dimensional (2D) grid configuration over a crevasse field at an alpine glacier in Switzerland (see Fig. 1). We use this dataset to investigate whether we can use this novel seismic instrumentation to quantify previously unquantifiable subsurface ice fabric damage and interrogate the possible role of fluids in fracture mechanisms in more detail than previously possible.

## RESULTS

Icequakes detected at the Gornergletscher crevasse field are shown in Fig. 1B. We detect 1355 icequakes within the vicinity of the array from 17 to 23 October 2023. The icequake detection rate through time is shown in Fig. 1C. Icequake rates are higher when surface temperatures are above freezing, with the exception of an anomalously active 6-hour period on 20 October after an extreme storm that even forced us to stop operating the DAS interrogator for safety reasons. This storm episode and the other peaks in icequake activity suggest an ∼12-hour lag between peaks in temperature or precipitation and icequake activity. This lag is likely caused by a buildup of water at the crevasse field that drives hydrofracture and/or glacier speed up.

Most of icequakes are thought to originate from near the glacier surface because they generate strong surface waves, as shown by the signals from a typical event in Fig. 1D. As well as surface-wave phase arrivals, the icequakes typically have smaller amplitude *P*-wave arrivals but no discernible *S*-wave arrivals. Our confidence in a lack of *S*-wave energy is based on the characteristic Rayleigh wave–envelope nature of the surface-wave arrivals and successful back-migration of energy through isotropic literature-informed *P-* and surface-wave velocity models (*28*) resulting in a single coherent icequake source location. This observation provides us with additional confidence that events might be driven by dominantly volumetric failure rather than shear failure. One can also see where the fiber traverses crevasses from the seismic wavefield data (see relevant label, Fig. 1D). The lower-frequency signals observed here might be fluid resonance in the crevasses or excitation of the decoupled hanging fiber due to wind noise.

There is also a strong coda signal following the surface-wave arrivals. This could be caused by either fluid resonance within the crevasse actively fracturing (*29*) or seismic wave scattering off crevasses throughout the crevasse field. We back-migrate the energy of this coda for this example icequake and find that the energy originates from throughout the crevasse field, suggesting that the coda energy is dominated by scattering rather than fluid resonance (see the Supplementary Materials for further details).

**Fig. 2.**
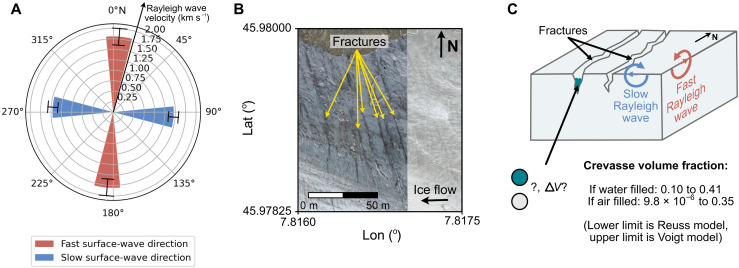
Surface-wave anisotropy tomography results. (**A**) Rayleigh (surface)–wave velocity inversion results, showing the fast and slow Rayleigh-wave velocities and azimuths from north. Uncertainty in velocity shown by black bars and the width of the colored bars denotes the azimuthal uncertainty (see the Supplementary Materials for the inversion misfit space used to estimate the uncertainties). (**B**) Enlarged aerial image of the crevasse field with some of the fractures labeled [with partially whitened section of image from satellite imagery as in Fig. 1, from the Swiss Federal Office of Topography (swisstopo)]. Imagery as in Fig. 1. (**C**) Schematic diagram showing the interpretation of the results in (A), along with the Reuss and Voigt effective medium theory bounds for the crevasse volume fraction calculated from the difference between fast and slow Rayleigh-wave velocities.

Because the dominant seismic energy generated by the icequakes is surface waves, a natural question that arises is: Can these surface waves be used to image crevasse fracture damage? Surface-wave energy is likely dominated by Rayleigh waves rather than Love waves because the surface waves are clearly observed on both the horizontally oriented DAS channels and the vertically oriented seismic nodes. Figure 2 shows results from a Rayleigh-wave velocity anisotropy inversion. The only parameters inverted are $v_{R,\text{fast}}$, $v_{R,\text{slow}}$, and ϕ, representing the fast and slow Rayleigh-wave velocities and the direction of the fast Rayleigh wave relative to north, respectively. We assume that ice velocity only varies with azimuth is otherwise homogeneous. $v_{R,\text{fast}}$ is found to be ∼1.8 km s^−1^, a physically plausible upper value for homogeneous ice (*28*). Similarly, the orientation of the fast Rayleigh-wave direction (red bar, Fig. 2A) is approximately parallel to the dominant strike of the surface crevasse expressions (see Fig. 2B), within uncertainty. The slow direction is, by definition, orthogonal to the fast direction and, hence, perpendicular to the crevasse fractures. If one assumes that the fast Rayleigh wave propagates through homogeneous ice, then one can infer that the slow Rayleigh-wave velocity is reduced by the presence of crevasse fractures. Effective medium theory can be used to estimate crevasse volume fractions and, hence, fracture damage. Effective medium theory is a way of describing how overall bulk medium properties vary when the composition of that bulk medium is varied. However, applying effective medium theory to describe the composite effect of multiple fractures is only valid if the fracture size or occurrence is smaller than one-third of the wavelength (*30*). We are in this regime (fracture spacing of ∼2 m for wavelengths of ∼17 m). For a crevasse-fractured medium, the bulk medium can comprise of a mixture of ice, air, and/or water. For simplicity, we assume that the medium comprises of ice and air only or ice and water only. We can then use the fast and slow Rayleigh-wave velocities to estimate the relative composition of the two components of the overall medium (*31*). In doing so, we assume that the fast Rayleigh wave is only sensitive to the velocity of ice, whereas the slow Rayleigh wave is sensitive to crevasse fluids (air or water) and ice [see Fig. 2C for schematic description, with a theoretical description provided in Materials and Methods (the “From seismic velocity to crevasse volume fraction” section)]. If crevasses are water filled, then the crevasse volume fraction ranges from 0.10 to 0.41. If the crevasses are air filled, then the crevasse volume fraction ranges from 9.8 × 10^−6^ to 0.35. The lower and upper ranges correspond to Reuss and Voigt effective medium parameterizations, respectively. Note that we do not know the extent to which fractures are air or water filled and cannot constrain their connectivity (*32*). Field observations indicate that the crevasses are likely air filled, at least to visible depths (2- to 3-m depth from surface). Although the air-filled crevasse volume fraction range is four orders of magnitude, we tentatively suggest that the crevasse volume fraction lies at the upper end because the crevasse surface expressions are sufficiently large that we deem it fair to assume that the fractures elastically behave predominantly in series, corresponding to a Voigt effective medium formulation. In any case, seismic-wave velocities are clearly highly sensitive to crevasse damage, with seismic velocity anisotropy variations of ∼20%. For context, this magnitude of anisotropy is two to four times greater than that observed in the entire of Earth’s mantle (*33*) and comparable to the greatest anisotropy observed during volcanic eruptions where rock exceeds fracture criticality (*34*). Our results are in contrast to glaciers covered in a firn layer, where negligible anisotropy has been observed (*35*).

**Fig. 3.**
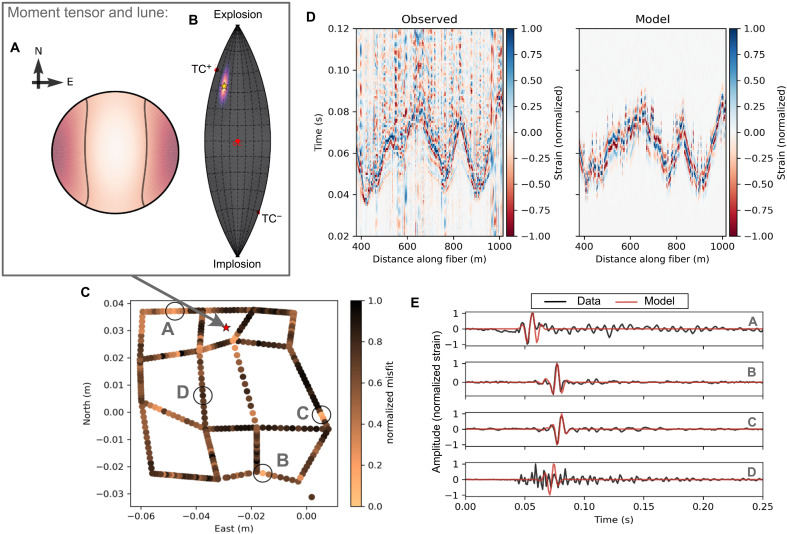
Example moment tensor inversion result for one icequake. (**A**) Upper hemisphere focal mechanism solution (red denotes compressional first arrivals, and blue denotes dilatational first arrivals). (**B**) Lune plot for the icequake. Colors denote the probability density distribution associated with the result. (**C**) Data-model misfit for all DAS channels plotted spatially. (**D**) Observed (data) wavefield, optimal modeled wavefield, and difference between the two. All amplitudes are normalized. (**E**) Examples of waveform observations for four DAS channels, corresponding to labels in (C).

While surface waves provide insight into fracture damage extent, icequake source inversion can provide insight into the fundamental fracture mechanisms that generate and extend the crevasses. Results for a single crevasse icequake are shown in Fig. 3. The optimal moment tensor result is plotted in Fig. 3A, showing volumetric opening in the approximately east-west (E-W) direction. The mechanism type is confirmed in lune space in Fig. 3B, lying 70% of the way from a double-couple (DC) source to a positive opening tensile crack. This, combined with the opening orientation, is consistent with what we would hypothesize for a crevasse icequake at the study site. The small proportion (∼30%) shear (or DC) component is perhaps unexpected. Some of this shear component could be due to uncertainty in the posterior solution but Fig. 3B suggests that, even after accounting for uncertainty, there is still a small DC component. Similar results have previously been made for alpine crevasses icequakes (*22*). We interpret this to be due to glacier flow not being entirely perpendicular to crevasse fracture, causing a horizontal shear component in the subsurface stress field. However, the mechanism of crevasse failure does not necessarily have to reflect the local stress field (*36*). We would suggest though that the shear is not associated with gravitationally driven flow of the glacier itself because the most likely plane of shear would be in the plane of the crevasse, which is perpendicular rather than parallel to bulk ice motion. Figure 3B also clearly indicates the benefit of many DAS channels, offering spatially dense measurements that also cover a 360° range of back-azimuths, significantly reducing the uncertainty in moment tensor space compared to previous studies using less than ten seismometers (*9*). The small uncertainty in moment tensor mechanism is due to the generally high quality of fit of many of the DAS channels. The data misfit for every DAS channel is shown in Fig. 3C, with the entire observed and modeled wavefield shown in Fig. 3D. Figure 3E shows the waveforms for four DAS channels in detail. Both *P*- and surface-wave phase fits are approximately noise limited (i.e., have the lowest possible misfits) for channels near (A) and far (B and C) from the icequake source for both fiber channels oriented E-W (A and B) and north-south (C). However, some channels, especially those on the more heavily fractured eastern side of the crevasse field, show higher misfits. An example of this is the waveforms labeled (D) in Fig. 3E. We interpret these poor misfits to be caused by strong coda energy, with the modeled surface waves being incorrectly time shifted to some arbitrary point in the coda. However, overall, although these channels detrimentally affect the inversion result, the moment tensor inversion remains constrained by channels with lower misfit from a wide range of back-azimuths and hypocentral distances.

**Fig. 4.**
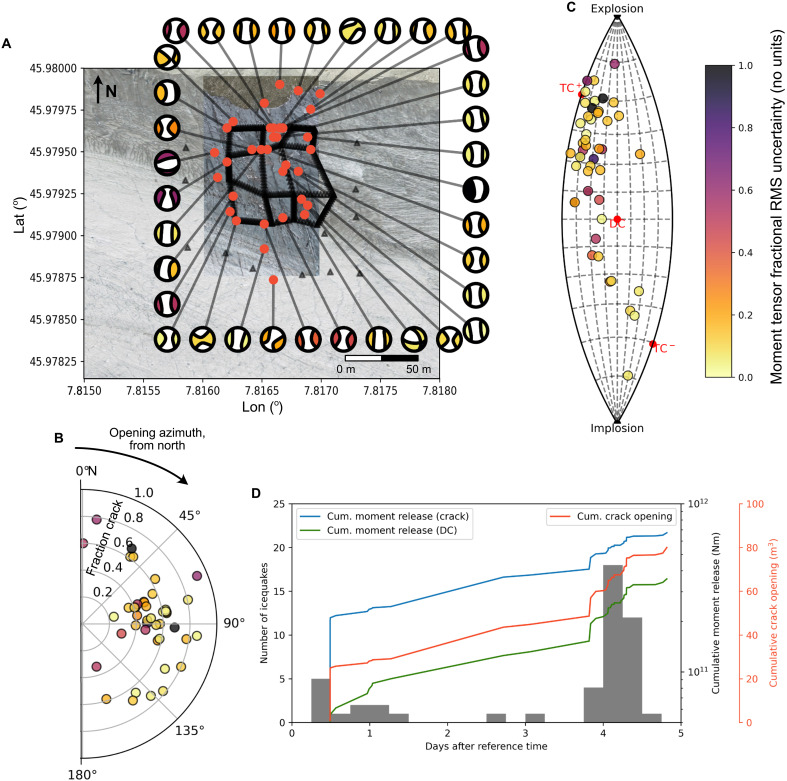
Summary of icequake moment tensor results for 50 icequakes. (**A**) Map plot showing focal mechanisms for the 50 icequakes for which full-waveform moment tensor inversion could be performed, colored by uncertainty [see (B) for scale]. (**B**) Crack component fraction for all the icequake moment tensor results with opening azimuth. (**C**) Lune plot of all events, showing proportion of isotropic, crack, and DC components. (**D**) Cumulative moment release (separated as DC and crack contributions) and estimated crack opening volume through time. RMS, root mean square.

Icequake moment tensor results for icequakes of sufficient signal-to-noise ratio (SNR) (those that exhibit clear *P*- and surface-wave arrivals on >50% of receivers) are shown in Fig. 4 (50 icequakes). These moment tensors are found via full-waveform inversion using data from both the fiber optics and node instrumentation (for details, see the “Full-waveform source mechanism inversion” section). The source mechanism types dominantly lie near the pure opening tensile crack region (labeled ${\text{TC}}^{+}$) of the lune, although most events exhibit some component of shear (Fig. 4B). There are a few anomalous events that lie between a DC and closing tensile crack regions. Some of the events lying nearer the DC region than closing tensile crack region have higher uncertainties (>40%), yet some of the events nearer the pure closing tensile crack region (labeled ${\text{TC}}^{-}$) have low uncertainties (<10%). The moment tensor orientations are consistent with approximately E-W opening of crevasses (see Fig. 4). One example of a closing crack is shown in the south-west corner of the crevasse field, where it may be possible that fractures could close because, downstream of this point, there are no visible surface fractures. However, these few closing tensile crack source inversion results should be treated with caution as they typically exhibit higher waveform misfits and are dominated by fitting surface waves rather than dilatational *P*-wave arrivals (see fig. S4 in the Supplementary Materials). This is generally a challenge for full-waveform inversion with body and surface waves, whereby the surface waves typically have higher amplitudes that can dominate the misfit yet hold less information than body waves for source mechanism inversion. This is partially associated with free-surface effects limiting the resolvability of isotropic moment tensor components (*37*), although the dense focal sphere coverage provided by fiber-optic sensing may minimize this limitation. Furthermore, we do not observe clear dilatational *P*-wave first arrival polarities that would provide conclusive evidence of closing cracks (*37*).

The full-waveform source inversion also provides estimates of icequake size, which can be used to calculate opening volumes. The cumulative seismic moment release of both the crack and DC components of the icequakes through time is shown in Fig. 4D. The total icequake crack opening volume of the 48 events combined is 84 m^3^, with the largest opening for a single event being of the order of 2 m^3^, for the icequakes included in Fig. 4. This only captures part of the total crevasse opening volume from the entire dataset. Assuming that the icequakes for which we could not obtain moment tensor solutions are all smaller in magnitude than those in Fig. 4, the minimum observed icequake opening volume (0.002 m^3^) is an upper limit for the remaining 1307 icequakes. Assuming that the remaining icequakes are at this upper limit gives a total crevasse field opening volume of ∼87 m^3^ for all the icequakes combined. This is only ∼0.1% of the total crevasse field volume. However, this opening only occurred over 5 days. If we assume that this rate of opening occurs throughout 1 year (of the order of the assumed time taken for ice to migrate over through the entire crevasse field region, ∼50 m year^−1^), then it would equate to ∼8.4% of total ice volume, which is the same order of magnitude as that estimated by the surface-wave anisotropy analysis (assuming a Voigt model dominates). However, these estimates of opening volumes are larger than those found previously in the literature, which were of the order of 100 cm^3^ (*38*). This could be due to uncertainty, with the moment tensor fractional uncertainty of some icequakes nearing 100% (see Fig. 4C) or an overestimate of seismic efficiency (assumed 100% efficient here), but, given the agreement with the surface-wave anisotropy results, we favor the interpretation that it may just be an extremely active crevasse field.

## DISCUSSION

### Key findings at Gornergletscher

**Fig. 5.**
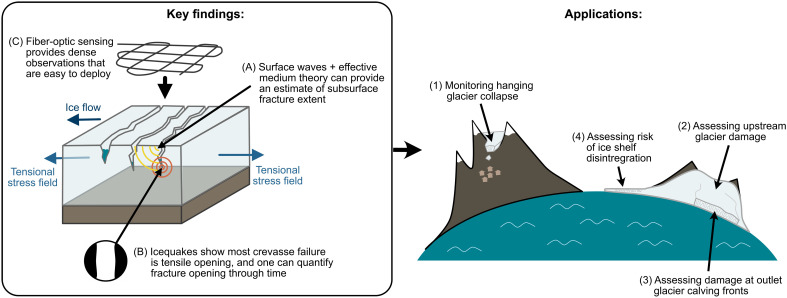
Summary of key findings of this study and future applications of fiber-optic sensing technology for fracture damage monitoring at glaciers.

Crevasse fields are seismically active environments (*9*, *22*, *25*, *26*). The field site studied here is no exception, generating >1000 icequakes in 7 days that can be used to assess fracture damage extent and mechanisms. Existing subsurface fracture orientations and extent can be estimated using surface-wave energy, with either air- or water-filled fractures resulting in similar fracture volume upper bounds [key finding (1), Fig. 5]. Previously unidentified fracture damage can be illuminated using icequake source inversion [key finding (2), Fig. 5]. As anticipated, we find that icequakes are generally dominantly generated by opening tensile crack failure, with the orientation of opening consistent with glacier flow–driven surrounding stress conditions. However, almost all events contain some shear component. This is consistent with the glacier flow regime, where there is likely some component of horizontal and/or vertical stress field perpendicular to ice flow. Such shear components have been observed before at Gornergletscher (*22*). Intriguingly, a few icequakes exhibit closing tensile crack mechanisms. Although the location of such icequakes is plausible (at the south-western corner of the crevasse field where crevasses visibly start to close), we are not confident in this result because the inversion fits are dominated by surface waves rather than dilatational body waves. However, if such closing crevasse icequakes do occur, then the only hypothesized mechanism in the literature is sudden fluid evacuation (*9*), although the plausibility of this is an open question. Using DAS and node amplitude information, we can also estimate seismic energy release. Quantifying seismic energy release (or moment) allows us to estimate crevasse opening volumes during failure. Cumulatively, these opening volumes account for approximately the same fracture opening extent observed by the surface-wave tomography, assuming an average failure rate consistent over the duration that the ice takes to traverse the crevasse field. This finding suggests that fracture, rather than subsurface melt, may dominate subsurface volume changes at temperate alpine glaciers. The far greater sampling density provided by fiber-optic sensing compared to conventional instrumentation (100× more sensors), combined with the speed and ease of deployment (hours rather than days and traversing crevasses), suggests that fiber-optic sensing is a useful tool for quantifying fracture damage extent and fracture generation [key finding (3), Fig. 5].

### Implications for subsurface glacier fracture monitoring

The results presented here are relevant for quantifying and/or monitoring subsurface fracture damage at other glacier systems beyond Gornergletscher. A summary of possible applications is shown in Fig. 5. Perhaps the most pertinent example could be monitoring unstable hanging glaciers, where blocks of ice can suddenly calve off an alpine glacier terminus, posing a risk to communities living below (*39*, *40*). In 2022, 70,400 m^3^ of ice, rock and debris broke off Marmolada Glacier, Italy, killing 11 people (*41*). At the time of writing, an estimated 3,000,000 m^3^ glacier collapse in Blatten, Switzerland, just buried an entire village. Our results suggest that seismology, particularly distributed fiber-optic sensing, could provide a useful tool for assessing and monitoring the vulnerability of unstable hanging glaciers to suddenly collapse (if it can be deployed safely). Surface-wave anisotropy tomography can provide estimates of current fracture damage extent, while icequakes could be used to quantify further opening in real time. DAS could also measure quasistatic surface strain field dynamics too. Assuming that it is possible to calculate a maximum volumetric fracture opening threshold before catastrophic collapse, the techniques demonstrated in this work could provide a tool for estimating how close a glacier front is to failure. Fiber-optic cables can be deployed fast (order of hours) and provide real-time monitoring, assuming that a suitable power supply is possible and that fiber-ice coupling is comparable to that in this work. Rates of catastrophic glacier front collapse may accelerate in the future because of increased temperatures driven by climate change (*42*). However, this remains debated. In any case, new monitoring methods are important for contributing to future risk assessment and monitoring strategies.

The findings and approach presented here would also be directly applicable to globally pertinent ice sheets and ice shelves. First, our field site likely has far higher surface temperatures and, hence, meltwater generation than Greenland or Antarctica, yet subsurface volumetric opening appears to be dominated by fracture rather than water-induced melt even in this high-meltwater extreme. We therefore tentatively suggest that fracture-driven glacier subsurface damage logically should dominate in lower-meltwater instances too. Second, the orders-of-magnitude increase in seismic observations from DAS provides well-constrained estimates of present-day subsurface fracture damage and accurate estimates of real-time fracture opening. These estimates could be combined with remote-sensing derived surface fracturing (*10*) to calibrate a surface to subsurface fracture damage relationship. This could then be applied to estimate subsurface fracture damage at ice sheet or ice shelf scales. Such estimates could provide more reliable estimates of both ice shelf vulnerability to hydrofracture-driven disintegration (*7*) and a better understanding of how upstream outlet glacier damage might control calving rates into the ocean (*43*, *44*).

While our findings show promise for quantifying glacier fracture damage, there remain outstanding limitations, unknowns, and directions for future work. From a technical perspective, the high amplitude coda caused by scattering of the seismic wavefield from crevasses causes some receivers to spuriously contribute to full-waveform source inversion results. While, ordinarily, it would be straightforward to window only the seismic phases of interest, in this experiment, our receivers are so close to the source that isolating phases from coda is, in many cases, impossible. Similarly, both coda and surface waves make calculating seismic moment using traditional methods challenging (*45*), meaning full-waveform–derived seismic moments may be the only feasible method in such settings. One further technical challenge of note is practically deploying fiber-optic sensing in highly crevassed environments. For safety reasons, crevasses should ideally be snow free during deployment, yet to maximize fiber coupling, the fiber must therefore freeze into the ice rather than be snow buried. We suggest that this might limit deployments to a narrow time window in the transition from summer to winter, where snow is absent but temperatures drop below freezing overnight. Outstanding science challenges and future directions are as follows. First, how fracture damage upstream correlates with fracture damage downstream remains unknown. Whether fractures weld together or remain as preexisting weaknesses is an open question that is relevant to overall glacier stability. Second, our results emphasize the challenge associated with converting seismic velocity into fracture density. Effective medium models can result in a large range of possible fracture volume fractions, especially if the extent of fluid-filled fracturing is unknown. This highlights a need for theoretical developments in this area and likely more observations. A natural extension to this work, especially for regions with greater variations in vertical velocity structure, would be to use the ambient noise wavefield, as in (*35*, *46*), to image firn structure. The ambient noise wavefield contains a greater bandwidth spectrum of Rayleigh waves than our icequake surface-wave sources, meaning that one can interrogate variations in anisotropy with depth. One could extend such analysis further, using the envelope of the coda of surface waves and radiative transfer theory to quantify scattering attenuation and wavefield diffusivity in general (*47*), which could lead to more accurate quantification of fracture density. Furthermore, we perform a very simple surface-wave anisotropic velocity inversion but a more rigorous approach would be to perform an elastic full-waveform tomography inversion (FWI), describing the anisotropic medium using homogenization theory (where the concept that seismic waves see heterogeneities on scales smaller than the seismic wavelength as a frequency-dependent, homogeneous effective medium can be described in an FWI framework) (*48*). We also cannot quantify the extent to which fracture connectivity and geometry affects our surface-wave anisotropy measurements (*32*, *49*). Similarly, we assume that fractures rather than crystal alignment (*50*, *51*) dominate anisotropy. However, we deem the effect of any crystal preferred orientation anisotropy as minimal because the ice is repeatedly damaged upstream, in contrast to smoother flowing Antarctic ice streams. This assumption could be tested by numerically modeling fracture anisotropy (*52*). Last, we do not attempt imaging crevasse field fracture density in space or time, but this should be possible. Four-dimensional seismic velocity anisotropy tomography would be a natural extension beyond this work to understand crevasse field dynamics more comprehensively.

A final question that we briefly address is the contribution that DAS makes compared to the nodal sensors deployed. The DAS system costs approximately three times more than the nodal system and requires a continuous power supply, so the contribution of DAS should be at least briefly discussed. For detection and location of icequakes, the medium is approximately homogeneous, so the DAS and vertical-component nodes are both sensitive to body waves and surface waves. Although the nodes provide a higher SNR per channel, a previous study shows that the far higher number of DAS channels compared to nodes provides a greater contribution to detection and location than the nodes (*53*). However, this finding would likely not hold for strongly varying shallow velocity structures, where sensitivity to the horizontal versus vertical wavefield would play a more important role (*54*, *55*). Although DAS can be deployed in helically wound configurations to provide vertical and horizontal sensitivity (*56*), such deployments are typically both financially and logistically prohibitively expensive. For the surface-wave anisotropy, the nodes were not used in the analysis purely because we deemed the volume of DAS observations as sufficient to constrain the inversion. This is because the anisotropy inversion involves receiver-receiver cross-correlation, which is not typically limited by SNR in this case. While the nodes have a higher SNR than single DAS channels, in isolation, their sparser spatial sampling would have provided poorer azimuthal coverage and, hence, greater uncertainty in the inversion result. For the source mechanism inversion, the full-waveform approach allowed DAS and nodes to both contribute to the result in their native units. However, again, in this case, we anticipate that the nodes did not provide a substantial additional contribution compared to all the DAS channels combined because both DAS and nodes were sensitive to the *P*-wave and Rayleigh waves used. However, the full-waveform source mechanism inversion was also used to estimate seismic moment release, and, here, the nodes likely provided valuable additional constraint due to their higher SNR per channel. Practically, as previously mentioned, DAS has advantages for studying and monitoring glaciers. Deployment and retrieval of fiber-optic cables on glaciers can be more straightforward than nodes because, if the fiber melts in and then refreezes, then there are no tilt issues associated with nodes or seismometers (*57*). However, perhaps the greatest gain for monitoring is that a DAS interrogator provides a single point source of data recording and processing that could theoretically be telemetered for real-time monitoring.

Overall, we show that the high number of DAS observations (>400 receivers) provides quantification of fracture damage and mechanisms with acceptable levels of uncertainty. Although the limitations of effective medium theory limit our constraint of fracture damage extent, surface-wave anisotropy results are consistent with icequake fracture volume opening estimates. These results emphasize the potential of novel DAS technology for quantifying present-day fracture damage at any glacier system, with applications ranging from monitoring the vulnerability of hanging glaciers to catastrophic collapse to assessing fracture damage at globally pertinent ice sheets and ice shelves.

## MATERIALS AND METHODS

### Dataset

DAS and seismic node instrumentation were deployed at a crevasse field on Gornergletscher glacier, Switzerland. The experiment was undertaken from 17 to 23 October 2023. This time period was chosen to avoid crevasses being snow-covered and maximize the chance of meltwater being present in the system while also being cold enough for the DAS fiber-optic cable to melt and freeze into the ice on the surface, maximizing fiber coupling to the medium. The DAS system comprised a Sintela Onyx interrogator connected to a 1-km fiber-optic cable with a channel spacing of 1.6 m and a gauge length of 6.4 m. The sampling rate was 1000 Hz and the unit of measurement was strain. Twenty-nine Sercel WiNG nodes were also deployed (see Fig. 1). These nodes are micro-electrical-mechanical-system (MEMS) sensors, measuring vertical acceleration, with a sampling rate of 1000 Hz.

### Icequake detection and location

Icequakes are detected using the back-migration method, QuakeMigrate (*58*), which was recently adapted for use with DAS data (*53*). The method works by approximating the seismic energy arriving at receivers and stacks this energy in space and time to search for coherent seismic sources. The back-migration method is chosen because it performs well in high-noise environments and associates various seismic phases in a physics-informed way (*53*). Here, we use both *P*-wave and surface-wave phase information to detect and locate events. Icequakes are then relocated using NonLinLoc (*59*). The specific settings used for detection and location are given in Table 1. The velocity model used for both the back-migration and icequake relocation is a homogeneous velocity structure with a *P*-wave ($v_{P}$) and Rayleigh-wave ($v_{\text{Rayleigh}}$) velocities of 3.6 and 1.66 km s^−1^, respectively.

**Table 1.** QuakeMigrate settings for detecting icequakes in this study.

Dataset	Glacier
Phases used	*P*, surface
Sampling rate	1000 Hz
Frequency filter, *P*	10 to 250 Hz
Frequency filter, surface	5 to 150 Hz
Grid resolution, *x*	8 m
Grid resolution, *y*	8 m
Grid resolution, *z*	10 m
Short-term-average/long-term-average (STA/LTA) *P*	0.01/0.2
Short-term-average/long-term-average (STA/LTA) *S*	0.02/0.2
Coalescence detection threshold	1.15
Marginal window	0.25 s
DAS-specific settings:	
Spatial downsampling factor	1
Channel spacing	1.6 m
Gauge length	6.38 m
Semblance stacking	No

However, the glacier subsurface at the field site is highly fractured, likely resulting in an anisotropic velocity structure. In the following section, we describe a simple surface-wave tomography workflow used to refine icequake hypocenters further.

### Surface-wave velocity anisotropy tomography

Using the homogeneous and isotropic velocity structure described in the “Icequake detection and location” section results in anomalously high misfits for the full-waveform source inversion analysis performed in this study for certain DAS channel orientations. This systematic misfit bias is attributed to fracture-driven velocity anisotropy. To obtain a more accurate velocity model, we turn to Rayleigh-wave tomography. We are confident that the surface waves are dominantly Rayleigh waves because they are observable on the vertical-component seismic nodes as well as the DAS, so are vertically polarized. Ideally, we would have three-component node data to confirm characteristic elliptical Rayleigh-wave motion, but, unfortunately, we do not have such data. We set up an inversion scheme to iteratively invert for an azimuthally varying velocity structure and refined icequake locations asynchronously. Updating the (anisotropic) velocity structure and source locations iteratively is important because the velocity structure inversion is dependent on the source locations and vice versa. We iteratively update the tomography model and source locations until the reduction in misfit is negligible (<1%).

The data used for both the surface tomography and refined location inversions are differential surface-wave group-velocity arrival times between receivers derived from cross-correlation of the waveforms between receiver pairs. We use a 14-icequake subset of the entire dataset, manually selected on the basis of clear surface-wave arrivals to ensure sufficient azimuthal coverage (see fig. S2). A subset of the dataset is used to reduce noise in the results and reduce inversion computational cost. Differential arrival times are beneficial because surface waves have emergent arrivals (and are generally dispersive, although minimal dispersion is observed here due to the approximately homogeneous velocity structure in depth), so first arrivals cannot easily be identified and would represent a phase- rather than group-velocity measurement. To measure the differential arrival times between two receivers, we cross-correlate data windowed 0.05 s before to 0.15 s after the event origin time [similar to the approach in (*21*)]. Data are band-pass filtered between 10 and 500 Hz. We only retain receiver-pair measurements with a normalized cross-correlation value of >0.7. The data for every receiver pair that meet this criterion are combined into an overall data vector, $d^{\text{obs}}$.

For the Rayleigh-wave tomography inversion, we describe the velocity model structure using three parameters $m=\left(v_{\text{fast}},v_{\text{slow}},\phi \right)$, the speeds in the fast and slow directions (orthogonal to one another), and the angle of the fast-direction from north. Although we likely have sufficient observations to invert for a 2D laterally varying velocity structure, we opt to ensure that the model space is sufficiently small that we can perform the inversion via a grid search, allowing us to estimate uncertainty in a computationally efficient manner. We choose not to invert for a depth-dependent velocity structure because the crevasse-generated surface waves have a relatively narrow bandwidth (∼80 to 100 Hz). This approximately corresponds to having a maximum sensitivity to depths of ∼5 m below surface. The nonlinear forward model, G(m), which allows us to compute the synthetic data, is described as follows. For a given receiver, *i*, the arrival time is given by

$$t_{i}=t_{0}+\frac{d_{i}}{\left\|v_{i}\right\|}$$ (1)

where $t_{0}$ is the icequake origin time, $d_{i}$ is the source-receiver distance, and $\left\|v_{i}\right\|$ is the mean surface-wave velocity between the source and receiver, which is defined as

$$v_{i}=\left[\begin{array}{c} v_{\text{fast}}\text{cos}\left(\theta_{i}-\phi \right)\\ v_{\text{slow}}\text{sin}\left(\theta_{i}-\phi \right) \end{array}\right]$$ (2)

where $\theta_{i}$ is the back-azimuth from the source to the receiver relative to north. For two receivers, *i* and *j*, the differential arrival time is then given by

$$t_{j}-t_{i}=\frac{d_{j}}{\left\|v_{j}\right\|}-\frac{d_{i}}{\left\|v_{i}\right\|}$$ (3)

The forward problem is then defined as

$$G\left(m\right)=\left[\begin{array}{c} \frac{d_{2}}{\left\|v_{2}\right\|}-\frac{d_{1}}{\left\|v_{1}\right\|}\\ \frac{d_{3}}{\left\|v_{3}\right\|}-\frac{d_{1}}{\left\|v_{1}\right\|}\\ \vdots \\ \frac{d_{j}}{\left\|v_{j}\right\|}-\frac{d_{i}}{\left\|v_{i}\right\|}\\ \vdots \\ \frac{d_{n}}{\left\|v_{n}\right\|}-\frac{d_{n-1}}{\left\|v_{n-1}\right\|} \end{array}\right]$$ (4)

where *n* is the number of receivers (636 in our case). The misfit is quantified by the L2-norm between G(m) and $d^{\text{obs}}$. The model space is explored through a grid search to find the optimal model parameters, $m^{\text{opt}}$, and the associated uncertainty.

If the velocity model is updated, then the optimal icequake hypocenters will change. For surface waves, the source-receiver distance, $d_{i}$, is actually the epicentral distance because surface waves are generated from an interaction of seismic energy at the free surface. Therefore, if we simplify the location problem by inverting for icequake epicenters rather than hypocenters, then we can use the same forward operator as for the surface-wave tomography above (see Eq. 4). We simply instead invert for the model parameters $m=\left(x_{0},y_{0}\right)$, the epicentral location in km east and north. Similar to the tomography inversion above, our model space is small and so it is possible to efficiently perform a grid search to find the optimal model. Misfits are defined via the L2-norm as before.

#### From seismic velocity to crevasse volume fraction

The surface-wave velocity anisotropy can be used to estimate bulk crevasse volume fraction (the proportion of volume that is open, i.e., filled with air or water) using effective medium theory (*60*). For fractures to have an effect on seismic velocity anisotropy that can be described by effective medium theory rather than scattering, the fracture size or occurrence has to be smaller than one-third of the wavelength (*30*). We expect to be in this regime (for 100-Hz waves with a velocity of 1.7 km s^−1^, the wavelength would be 17 m, with fractures typically with separations less than 2 to 5 m). Numerous theories describe how to combine the effect of multiple media on seismic velocity, depending on the scale and geometry of cracks and the orientation of the waves relative to these heterogeneities. Here, we use two theoretical effective medium models that represent two extremes, providing minimum and maximum limits of bulk crevasse volume fraction. The lower limit is computed using a Reuss model and the upper limit is computed using a Voigt model (*31*). In both cases, we use the *P*-wave velocity, obtained by converting Rayleigh-wave velocities to *S*-wave velocities (assuming that ice is a Poisson solid) and *S*-wave velocities to *P*-wave velocities using a $v_{P}/v_{S}$ ratio of 1.95 (*51*).

The Reuss model assumes that elastic moduli act in parallel, describing a system where seismic-wave propagation is affected simultaneously by the various composite media. In this instance, the effective medium elastic modulus, $M_{\text{eff}}$, is given by (*31*)

$$\frac{1}{M_{\text{eff}}}=\frac{1-\zeta }{M_{\text{ice}}}+\frac{\zeta }{M_{\text{fluid}}}$$ (5)

where $M_{i}=v_{P,i}^{2}\rho_{i}$ where *i* is a particular material with a *P*-wave velocity, $v_{P,i}$, and density $\rho_{i}$. ζ is the crevasse volume fraction. The alternative is the Voigt model, which assumes that elastic moduli act in series, describing a system where seismic-wave propagation is affected sequentially by various composite media layers. In this instance, the effective medium elastic modulus, $M_{\text{eff}}$, is given by (*31*)

$$M_{\text{eff}}=\left(1-\zeta \right)M_{\text{ice}}+\zeta M_{\text{fluid}}$$ (6)

In both cases, $M_{\text{ice}}$ is defined as homogeneous and is related to the fast *P*-wave velocity (acting parallel to the crevasses), derived from converting the surface-wave tomography (as described above). $M_{\text{eff}}$ is defined as the elastic modulus in the direction perpendicular to the crevasses, which is related to the velocity in the slow direction from the surface-wave tomography. We calculate the volume fractions for both the Reuss and Voigt models for two fluids: air and water. The velocities and densities used for these fluids are: $v_{P,\text{air}},\rho_{\text{air}}=0.33\;\text{km}\;s^{-1}$, 1.2 kg m^−3^; and $v_{P,\text{water}},\rho_{\text{water}}=1.5\;\text{km}\;s^{-1}$, 1000 kg m^−3^. Substituting and rearranging the Reuss and Voigt equations provides us with lower and upper limits on crevasse volume fractions inferred from the surface-wave tomography results.

However, one should note that our effective medium approach is likely too simplistic to capture the full complexity of the system. More involved homogenization inversion methods (*48*) might allow one to use frequency dependent sensitivity information from the entire wavefield to constrain fracture fraction and extent. Furthermore, we have no constraint over the connectivity of fractures (*32*, *52*) or the geometry and length scale of fractures (*49*, *61*), both of which can cause variations in measured anisotropy.

### A note on using DAS amplitude information

To interrogate icequake fracture mechanisms directly, we perform full-waveform source mechanism inversions. Amplitudes of DAS channels relative to one another are essential for any source mechanism inversion, but, here, we also require absolute amplitudes because we aim to also invert for the seismic moment release of the icequakes via the source mechanism inversion. However, for this to be possible, one has to have an adequate understanding of the fiber coupling to the medium. To quantify this coupling, we use the method described in (*62*). This involves using multiple time windows of ambient noise as a reference wavefield and taking the scalar product channel by channel, which has been theoretically shown to provide an approximate representation of the relative coupling between channels (*62*). We then convert the relative coupling values into absolute values by assuming that at least one DAS channel is optimally coupled to the ice. Given that sections of the fiber were entirely frozen into the ice for most of the deployment, this is deemed a reasonable assumption. One should note that this method actually provides an estimate of coupling of the fiber to the medium and sensitivity to small (∼gauge-length) subsurface heterogeneities (*54*). However, for the purposes of this experiment, this approach is deemed sufficient.

### Full-waveform source mechanism inversion

The icequake source mechanisms presented in this work are found via a Bayesian probabilistic full-waveform inversion method [see (*63*) for full details on the method]. The observed data are windowed with 0.05 s before the arrival time and 0.2 s after the arrival time at each receiver. A band-pass filter between 25 and 150 Hz is applied, with the corner frequency of the icequakes typically being of the order of 100 Hz. We remove channels that are poorly coupled [see (*62*) for details] and those with turning points greater than 30° over a single gauge length.

The forward-modeled data are generated using the spectral-element method Salvus (*64*). One of the strengths of full-waveform inversion is that we can extract modeled data in the native units of each instrument (strain for DAS and acceleration for nodes), minimizing errors that would, otherwise, be introduced when converting between wavefields in different units. The velocity model used is that obtained by surface-wave tomography (see the “Surface-wave velocity anisotropy tomography” section for details). For each icequake source, the forward problem is solved six times, one for each unique component of the moment tensor. The inversion is performed using a least-squares method, with the uncertainty estimated via the covariance matrix associated with the solution. We allow small time-shifts to account for local 3D heterogeneities in the medium that are not captured by the surface-wave tomography model.

We automate the entire workflow so that we can process as many events as possible. While the location of each icequake is approximately known a priori, the spectral content of the icequake sources and attenuation path effects are not known. We therefore estimate the icequake source corner frequency and path-averaged attenuation before performing the forward model for each source individually using the spectral ratios method [see (*65*) for details]. This method takes the ratios of the observed spectra at each pair of receivers, which theoretically should contain the same source frequency content but different attenuation path effects. The corner frequency and the overall average attenuation value over all paths are used to constrain the forward model run for that particular event.

### Seismic moment and source volumes

In this work, we estimate seismic moment directly from the full-waveform moment tensor inversion (see the “Full-waveform source mechanism inversion” section), with the norm of the full moment tensor equal to the seismic moment, $M_{0}$. However, these estimates of seismic moment are dependent on the attenuation structure of the medium. As described previously, we use a spectral ratio method (*65*) to isolate attenuation path effects from source effects. The seismic moment estimates from the full-waveform inversion should represent the best estimates possible.

We also separate the seismic moment for each event into the fraction of the event that is DC and the fraction that is tensile crack. This deconvolution is arbitrary. We choose a DC-crack deconvolution simply because it best represents the physical system. We perform the deconvolution based on the description in (*66*). First, the eigenvalues of the moment tensor, M, $\lambda_{1},\lambda_{2},\lambda_{3}$ are calculated, where $\lambda_{1}>\lambda_{2}>\lambda_{3}$. Rotating the moment tensor into an arbitrary coordinate system, the crack and DC moment tensor components can be described by

$$M^{h,\text{DC}}=\left[\begin{array}{ccc} 0 & 0 & \sqrt{\left(\lambda_{1}-\lambda_{2}\right)\left(\lambda_{2}-\lambda_{3}\right)}\\ 0 & 0 & 0\\ \sqrt{\left(\lambda_{1}-\lambda_{2}\right)\left(\lambda_{2}-\lambda_{3}\right)} & 0 & 0 \end{array}\right]$$ (7)

and

$$M^{h,\text{crack}}=\left[\begin{array}{ccc} \lambda_{1} & 0 & 0\\ 0 & \lambda_{2} & 0\\ 0 & 0 & \lambda_{1}-\lambda_{2}+\lambda_{3} \end{array}\right]$$ (8)

One can then describe the total moment tensor by

$$M^{h}\left(\zeta \right)=M^{h,\text{DC}}+M^{h,\text{crack}}$$ (9)

$$=\left[\begin{array}{ccc} \lambda_{1} & 0 & \sqrt{\left(\lambda_{1}-\lambda_{2}\right)\left(\lambda_{2}-\lambda_{3}\right)}\\ 0 & \lambda_{2} & 0\\ \sqrt{\left(\lambda_{1}-\lambda_{2}\right)\left(\lambda_{2}-\lambda_{3}\right)} & 0 & \lambda_{1}-\lambda_{2}+\lambda_{3} \end{array}\right]$$ (10)

$$=\text{cos}\left(\zeta \right)M^{h,\text{DC}}+\text{sin}\left(\zeta \right)M^{h,\text{crack}}$$ (11)

This can then be used to estimate the volumetric opening of crevasse icequakes. For a volumetric source, we assume that the crack component of the moment can be equated to the volume of opening (or closing for a negative crack), ΔV, as follows

$$M_{0}^{\text{crack}}=|M^{h,\text{crack}}|=K_{\text{ice}}.\Delta V$$ (12)

where $K_{\text{ice}}$ is the bulk modulus of ice, taken to be ∼8.4 GPa here.
