# Supplementary material for: Quantifying subsurface fracture damage in glaciers using fiber-optic seismology
Source: Sci Adv. 2026 Jul 23;12(30):eaef1107. doi: 10.1126/sciadv.aef1107 (PMC13394404; doi:10.1126/sciadv.aef1107)
Supplement: Supplementary file 1 — Supplementary Text Figs. S1 to S4 [file sciadv.aef1107_sm.pdf]

Science Advances
AAAS

# Supplementary Materials for

## Quantifying subsurface fracture damage in glaciers using fiber-optic seismology

Thomas S. Hudson *et al.*

Corresponding author: Thomas S. Hudson, thomas.hudson@eaps.ethz.ch

*Sci. Adv.* **12**, eaef1107 (2026)
DOI: 10.1126/sciadv.aef1107

**This PDF file includes:**

Supplementary Text
Figs. S1 to S4

# Supplementary Materials

## 0.1 Source of icequake coda

Deciphering the origin of the energy after the body and surface wave arrivals is important for understanding the crevasse fracture dynamics at the study site. The signal could originate from fluid oscillations in the crevasse cavity (29), providing possible indications of fluid-driven faulting, or could be the result of energy scattered by the entire crevasse field. These hypothesizes can be tested by back-migrating the energy from the these signals back in time and space. If the energy originates from a locality approximately in the same location as the crevasse icequake source, then it is likely that the signal is due to fluid oscillations. However, if the energy originates from elsewhere, or is particularly diffuse compared to the icequake direct body and surface wave signals, then it is more likely dominated by scattering in the medium. Figure S1 shows the back-migration of energy through time for one icequake. The top panel shows back-migration from the body-wave onset, clearly defining the icequake epicentre. However, for different points within the coda, the energy clearly back-migrates to near the centre of the crevasse field. This suggests that the coda signal is dominated by scattering rather than providing information in support of fluid resonance. While we do not show examples for all icequakes, this example is typical of other icequakes in the dataset.

## 0.2 Surface wave anisotropy source-receiver distribution and misfit

Figure S2 shows the source-receiver distribution used for the surface wave tomography and Figure S3 shows the misfit space for the Rayleigh-wave velocity anisotropy tomography inversion result. The model space is 3D for the parameters $v_{fast}$, $v_{slow}$ and $\phi$, with Figure S3 showing slices through this model space that correspond to the optimal solution (the global minimum in the model space). The optimal solution and its associated estimated uncertainty are shown by the red star and red contour line, respectively.

## 0.3 Additional moment tensor solutions in detail

Figure S4 shows three more moment tensor solutions in detail. Each represents an arbitrarily chosen solution that represents an opening tensile crack (Figure S4a), a closing tensile crack (Figure S4b) and a double-couple solution (Figure S4c). We deliberately use the same set of DAS channels as in Figure 3 because they represent an unbiased sample of high SNR and low SNR (or high scattering) channels. See the main text for a further description of these channels. All events show a similar magnitude of uncertainty in lune space. However, only the opening tensile crack solution and double-couple solution provide good waveform fits on at least two of the four DAS channels (A,C) that should be expected. It is not feasible to show waveform fits for every DAS channel, but the majority typically have similar misfits to channels A and C. These results indicate that the opening tensile crack and near-double-couple results may likely be reliable throughout the entire dataset, but closing crack events should be treated with more caution.

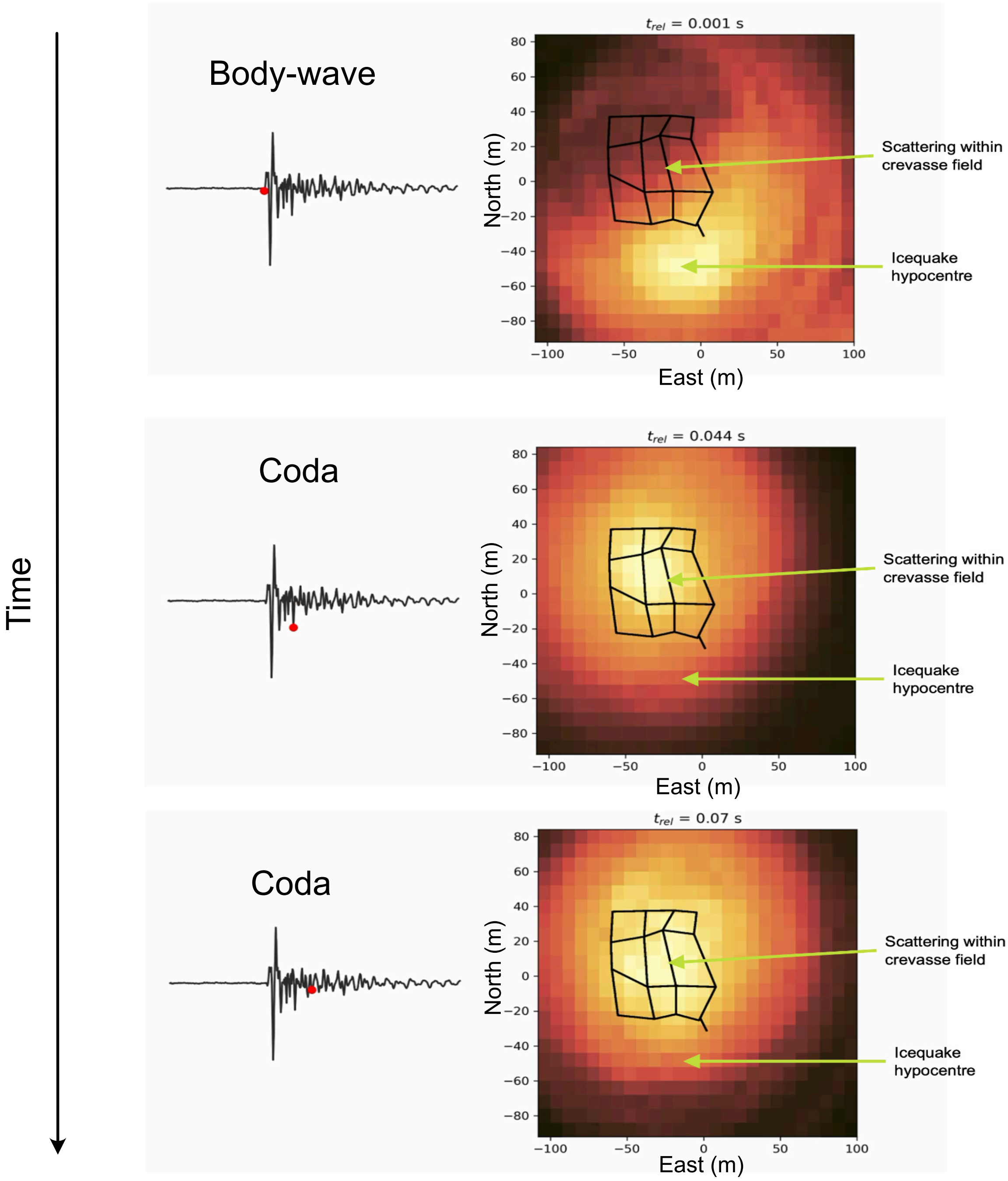


[H]

**Figure S1.** Figure for the example icequake shown in Figure 1, showing how the back-migrated energy looks from a body wave arrival and the subsequent coda. The seismic trace is from one DAS channel for illustrative purposes only, with the back-migration performed on all DAS channels simultaneously. Back-migration performed using QuakeMigrate (53). Heat maps show normalized back-migrated energy for that snapshot in time for all receivers stacked. Hotter colours indicate higher back-migrated energy values.

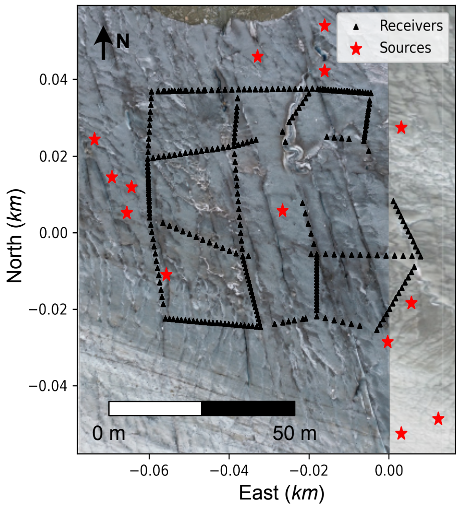


[H]

**Figure S2.** Source-receiver distribution used for the surface wave tomography. Detailed background imagery of section of the study site is from UAV imagery obtained during the deployment, overlaid on 2023 aerial imagery from the Swiss Federal Office of Topography (swisstopo).

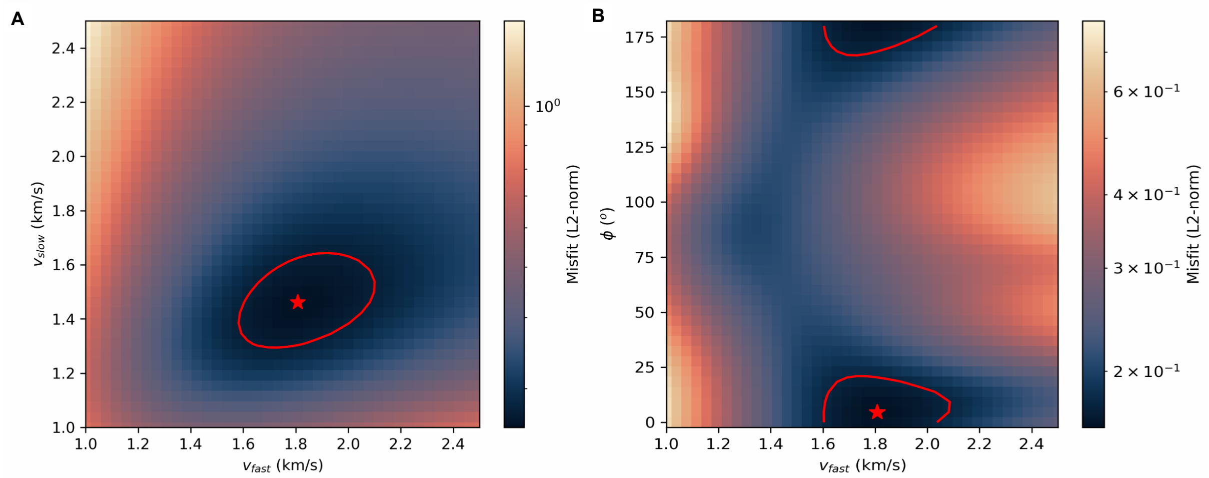


[H]

**Figure S3.** Surface wave tomography misfit space by model parameter. a. $v_{fast}$ vs. $v_{slow}$ model parameter space, sliced at the optimal value of $\phi$ ($4^o$ from north). b. $\phi$ in degrees from north vs. $v_{fast}$. Misfit amplitudes are for an L2-norm misfit function used in the analysis. Red stars show global minimum and red contour lines represent estimate of uncertainty in the result.

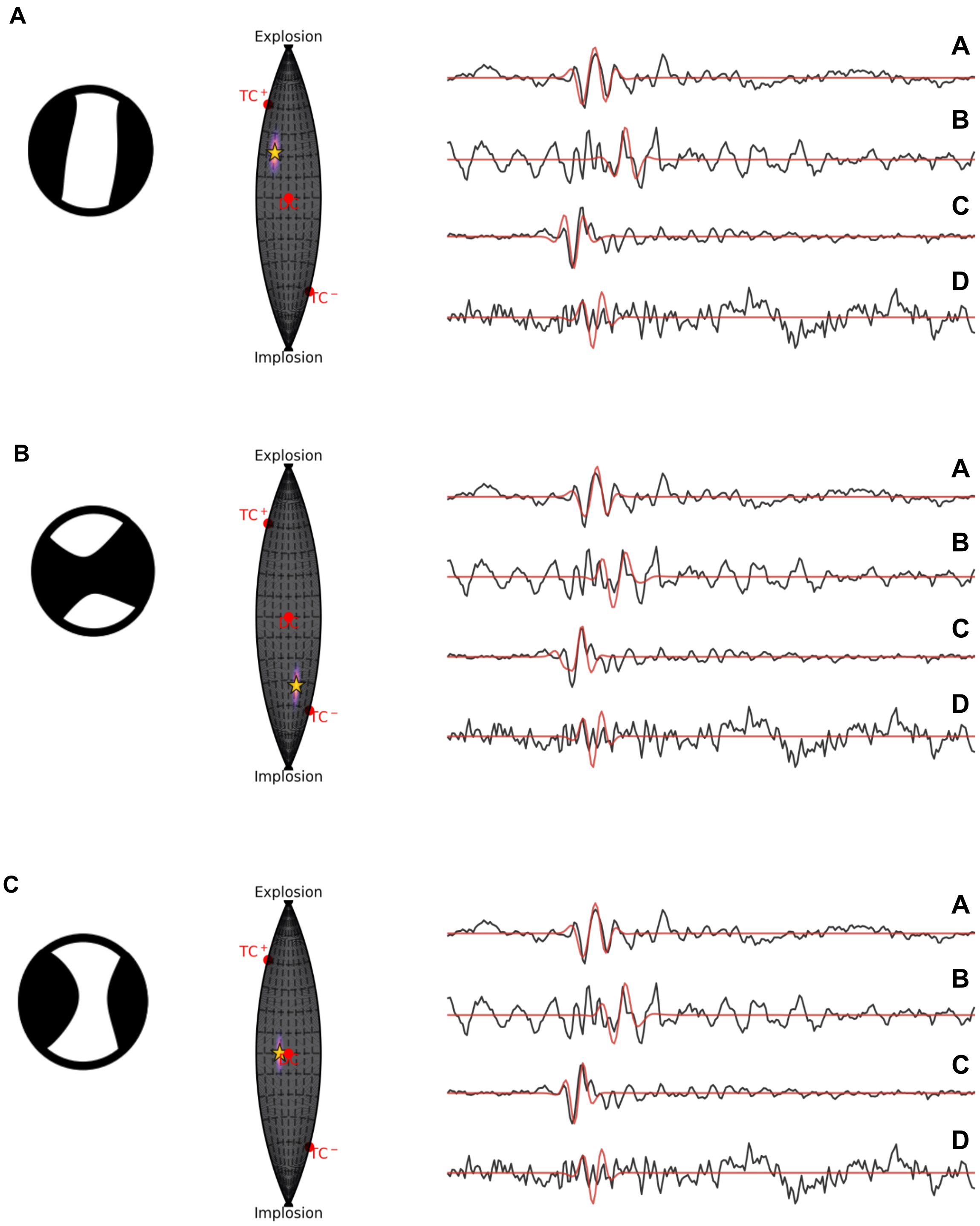


[H]

**Figure S4.** Examples of other moment tensor solutions in detail, including the optimal focal mechanism solution, a lune plot including the associated uncertainty in the solution, and four DAS channel modelled (red) vs. observed (black) waveforms. a. Example of icequake falling between a pure tensile opening crack and DC solution. b. Example of a pure closing tensile crack. c. Example of a pure DC solution. Labels A-D are the same as in Figure 3 in the main text.
